# The Impact of Salvage Radiotherapy in Recurrent Endometrial Cancer: A Review Focusing on Early-Stage, Endometrial Cancer Locoregional Relapses

**DOI:** 10.3390/life15071013

**Published:** 2025-06-25

**Authors:** Emmanouil Maragkoudakis, Theodoros Panoskaltsis, Kitty Pavlakis, Maria Grenzelia, Evangelia Kavoura, Georgios Papageorgiou, Ioannis Georgakopoulos, Andromachi Kougioumtzopoulou, Efrosyni Kypraiou, Nikolaos Trogkanis, Evangelos Maragkoudakis, Vassilis Kouloulias, Anna Zygogianni

**Affiliations:** 1 Department of Clinical Radiation Oncology, Attikon University Hospital, Medical School, National and Kapodistrian University of Athens, 11528 Athens, Greece; 2 Radiation Oncology Centre, Iaso General Clinic, 15123 Athens, Greece; 3 Second Department of Obstetrics and Gynecology, Aretaieion University Hospital, Medical School, National and Kapodistrian University of Athens, 12462 Athens, Greece; 4 K Pathology Department, IASO Women’s Hospital, 15123 Athens, Greece; 5 Second Medical Oncology Department, Iaso General Clinic, 15123 Athens, Greece; 6 Radiation Oncology Unit, Aretaieion Hospital, School of Medicine, National and Kapodistrian University of Athens, 11528 Athens, Greece

**Keywords:** uterine cancer, endometrial cancer recurrence, salvage radiotherapy, salvage brachytherapy, vaginal relapse, novel treatment strategies

## Abstract

Background/Objectives: Definitive radiotherapy (RT) is a frequently employed salvage option in early-stage, endometrial cancer (EC) loco-regional recurrence patients. Local control (LC) and survival rates are highly variable in the literature. The aim of this review is to assess the impact of modern salvage radiotherapy (SRT) in this group of patients. Methods: A systematic review of the literature was performed, focusing on studies that included EC local recurrence patients receiving SRT after 2000 to reflect advances in radiotherapy techniques. Our report followed the principles as outlined in the preferred reporting items for systematic reviews and meta-analyses (PRISMA) statement. Nine studies were included in our analysis with a total sample size of 648 patients. Conclusions: SRT offers excellent LC rates in this group of patients with minimal ≥ grade 3 toxicity. Salvage rates are limited by the presence of well-known risk factors for loco-regional relapses, with distant control being the primary mode of failure, resulting in lower survival rates. The decision to omit adjuvant RT should be weighed against the anticipated salvage outcomes in case of relapse.

## 1. Introduction

Endometrial cancer is the commonest gynaecological malignancy in the Western World. In the United States of America (USA), approximately 67,880 new cases were recorded in 2024, accounting for 3.4% of all new cancer cases, and the incidence is increasing [1]. Most cases are diagnosed at an early stage, and the mainstay treatment is surgical, total abdominal hysterectomy and bilateral salpingoophorectomy (TAH and BSO) through minimally invasive approaches. In most cases, early diagnosis, in combination with favourable histopathological markers, portent an excellent prognosis, with an estimated five-year overall survival of 80.8% [1].

Adjuvant treatment following surgery is traditionally based on clinicopathological risk factors, and more recently, molecular status has been integrated in the risk stratification groups, as per the ESGO-ESTRO-ESP 2021 guidelines and FIGO staging 2023 [2,3]. Low-risk patients need no adjuvant treatment as, following TAH and BSO, the local control and overall survival is excellent [4]. Adjuvant radiotherapy (RT) is employed in intermediate, intermediate-high and high-risk patients as it improves loco-regional control [5,6,7].

However, in practice, the decision to administer adjuvant radiotherapy is highly variable. The main reason is that, despite its proven benefit on local control, there is no positive effect on survival [5,6]. In addition, radiotherapy comes at a cost of acute and late side effects, which could negatively impact the quality of life. This is, mostly, the case with adjuvant external beam radiotherapy (EBRT), as opposed to vaginal brachytherapy (VBT), which appears to be less toxic [8].

For this reason, many argue against the use of adjuvant radiotherapy and limit its role in salvage settings. Also, this argument is mounted on excellent local control rates that salvage radiotherapy (SRT) offers, as shown in the PORTEC-1 control group, where a complete response was 89% for the radiotherapy-naïve patients that were salvaged following vaginal recurrence [9]. This has led the Nordic countries to omit adjuvant radiotherapy in most endometrial cancer patients, taking into account cost-effectiveness parameters [10,11]. Therefore the question whether salvage radiotherapy instead of adjuvant is a valid treatment option for most patients with risk factors for loco-regional relapse has yet to be addressed. The first step to answer the question is to assess the impact of salvage radiotherapy (SRT) on local control (LC) and overall survival (OS), which is the aim of this review.

## 2. Materials and Methods

**Figure 1:**
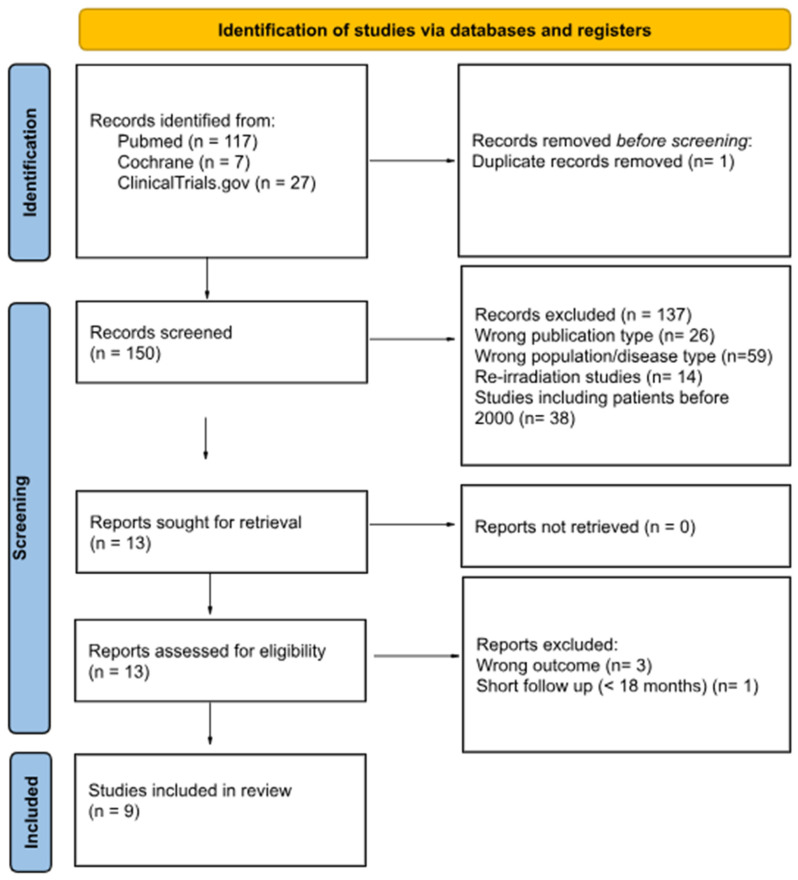
PRISMA 2020 flow diagram.

Search Methods: A systematic review of the literature was performed in PubMed, the Cochrane library and ClinicalTrials.gov. Studies published in the English language from peer-reviewed journals were included up to 21 March 2025. Studies with patients receiving salvage RT before 2000 were excluded to assess the impact of modern radiotherapy techniques [intensity-modulated radiotherapy (IMRT), volumetric arc therapy (VMAT) and image guided adaptive brachytherapy (IGABT)] and to reflect practice as set by the landmark trial PORTEC-1. Our report followed the principles as outlined in the preferred reporting items for systematic reviews and meta-analyses (PRISMA) statement (Figure 1) [12].

Search terms included “endometrial cancer recurrence”, “endometrial cancer relapse”, “salvage radiation therapy” and “salvage brachytherapy”.

The population type included Early-stage, endometrial cancer (EC) patients presenting with loco-regional relapse (LRR) who received definitive salvage radiotherapy (SRT). Loco-regional recurrence includes vaginal and/or pelvic/paraaortic relapses. Re-irradiation studies were excluded. Studies with patients who had received adjuvant RT following surgery were not excluded, provided they represented the minority, and, in this case, our focus was on the results of the RT-naïve subgroup.

Outcome types: Studies must include outcomes on local control and overall survival and must have a minimum median follow-up of 18 months.

## 3. Results

### Number and Type of Studies

Nine studies with published reports were included. The year of publication ranged from 2013 to 2024. One was a phase III trial, and the other eight were retrospective studies. All studies reported local control results, whereas overall survival and toxicity were not reported in two studies, respectively. A summary of the literature can be found in Table 1.

The first retrospective study was by Lee et al. from Harvard Medical school [13]. They reviewed 44 patients who received SRT due to endometrial cancer vaginal recurrence and were treated with 3D image-guided adaptive brachytherapy (IGABT), 80% of which had interstitial BT (ISBT). The majority (70%) had received no prior adjuvant radiotherapy, as they were FIGO stage I and had low-grade endometroid histology (73%). All RT-naïve patients underwent a combination of EBRT and vaginal BT. A total of 14% presented with simultaneous nodal recurrence. The mean cumulative dose in EQD2 was 75.5 Gy (EBRT and BT). With a median follow-up of 24 months, they reported the following 2-year outcomes. The local control (LC), disease-free survival (DFS) and overall survival (OS) of the RT-naïve group were 96%, 72% and 80%, respectively. A total of 50% (8/16) of failures were due to distant metastases. Based on univariate analysis (UVA), a high histological grade, distal vagina recurrence and a low cumulative dose were associated with local failure. Based on multivariate analysis (MVA), advanced age and a high histological grade were negatively associated with DFS and advanced age with OS. MVA was not performed for LC due to a limited number of events. Only one RT-naïve patient (3%) experienced grade 3 late toxicity.

Vargo et al., from the University of Pittsburgh, retrospectively reported the outcomes of 41 EC local recurrent patients [14]. A total of 80% presented with isolated vaginal recurrence, while 20% had vaginal and regional recurrence at presentation. None of them had received prior adjuvant RT, as 83% were initially Stage I and 79% had a low histological grade. All patients, except one, received a combination of EBRT and vaginal BT, and in the vast majority of those who had EBRT (90%), the IMRT technique was used. The median cumulative EQD2 dose was 76 Gy (D90-CTV). With a median follow-up of 18 months, 3-year LC, distant control (DC), DFS and OS were 95%, 61%, 68% and 67%, respectively, with the distant failure being the leading cause of failure. The size of the vaginal lesion was the only significant predictor of local failure based on UVA. Based on MVA, the depth of myometrial invasion remained a significant predictor of DC and OS. Grade 3 late toxicity was limited to 5% of patients. (One patient developed vaginal necrosis, and one patient had ileus complicated by a perirectal abscess and fistula.)

Chapman et al. retrospectively reviewed their experience from the University of California, San Francisco [15]. They included 30 EC recurrent patients with isolated vaginal recurrence (none with regional) and no prior adjuvant RT or systemic therapy. This cohort was at a fairly low risk for recurrence, with 86% being initially stage I and 63% having a low grade. All had a combination of EBRT and vaginal BT, with 90% receiving ISBT. The median cumulative EQD2 dose was 68.25 Gy with a median D90-CTV achieving higher levels (70.83 Gy). With a much longer median follow-up (76.4 months), their published 5-year outcomes are as follows: loco-regional failure-free survival (LRFFS), progression-free survival (PFS), distant failure-free survival (DFFS) and OS were 87%, 75%, 86% and 77%. MVA was not reported due to the small number of events. Based on UVA, a high histological grade and stage >IA were associated with worse OS and DFFS. One patient died from small bowel obstruction (SBO), with toxicity potentially related to the treatment, 11 months post-salvage RT. No other ≥Gr 3 toxicity was documented.

Arden et al. published their experience from Michigan, including 28 recurrence patients (82% isolated vaginal, 14% pelvic nodal and 4% vaginal and pelvic at presentation) who received SRT [16]. At the initial diagnosis, most of them were in the early stage (stage I: 93%) and had a low grade (79%). None had received adjuvant RT, and 96% had EBRT and vaginal BT combined treatment. The median cumulative EQD2 dose was 67.6 Gy. With a median follow-up of 21 months, 2-year LC, DFS and OS were 93%, 80% and 88%. UVA/MVA could not be performed. No grade 4–5 toxicities were seen.

The next retrospective study came from Houston, including 30 patients with mostly isolated vaginal recurrences (7% vaginal and pelvic) [17]. Sapienza et al. did not exclude patients with prior adjuvant RT, though none had received EBRT, and only one had vaginal BT; despite that this cohort was represented more frequently by higher-risk patients (Stage I: 63% and low grade: 63%). The combined EBRT/VBT treatment was received by 80%. The median cumulative dose was calculated using the EQD2 formula, yet it was not reported as such. Chemotherapy with RT was given to 70% of patients. With a median follow-up of 53 months (4.4 years), the 5-year rates of LC, regional control (RC), metastasis-free survival (MFS), DFS and OS were 89%, 91.5%, 75.5%, 69% and 83%, respectively. Based on UVA, nodal recurrence at presentation was associated with worse survival compared to vaginal only (50% vs. 85.9%). Factors associated with improved DFS were endometrioid histology, time to recurrence >9 months, combined EBRT and VBT and an EQD2 dose of >75 Gy, with the latter two being treatment-related and therefore potentially modifiable factors. Chemotherapy was given concurrently with SRT to 67% of patients but without any statistical improvement in DFS-OS. 5-year late toxicity was reported: rectal bleeding: 31%, SBO: 18% and pelvic insufficiency fracture: 13%.

Lindemann et al. reported their population-based experience from Norway, including 139 RT-naïve patients presenting with isolated vaginal relapses [18]. This is the largest cohort published in the literature due to the high number of patients omitting adjuvant RT according to the Norwegian national guidelines [19]. Most of the patients were stage I (75%), and among them, the majority were either at a low or intermediate risk (63%). Combined EBRT/VBT was delivered to 62% of patients, while the other 38% had the boost dose delivered at relapse with EBRT instead of VBT. The median cumulative EQD2 dose was 70 Gy. The median follow-up was 79 months (6.6 years), and 5-year and 10-year DFS and OS were reported: DFS: 65% at 5 and 37% at 10 years and OS: 68% at 5 and 38% at 10 years. A total of 40% developed a second relapse, with the majority being outside of the irradiated field (74%). Based on MVA, stage I high-risk, stages II-III and EBRT patients without VBT were all significantly associated with increased risk of a second relapse and death. There was no report on toxicity.

The second largest retrospective analysis comes from another Nordic country, Denmark. Ørtoft et al. reported the loco-regional recurrence patterns and salvage rates in a Danish population that did not receive post-operative radiation therapy, aiming to analyse the impact of omission of adjuvant RT as a national strategy, rather than estimating the salvage RT rates and post-SRT survival [20]. Among the initial cohort of 3723 patients with endometrial cancer treated with surgery, 121 isolated vaginal and 20 isolated pelvic recurrences treated with definitive salvage RT were identified. With a median follow-up of 109 months (9.1 years), the crude local control rate was 91% for isolated vaginal recurrence and 70% for pelvic recurrence. No survival outcomes were reported, as this was not the aim of the study. However, the investigators reported that among the 121 vaginal recurrence patients salvaged with RT, only 5.8% died of an unsuccessfully treated vaginal relapse, while 23.1% died due to disseminated disease and 22.3% from non-cancer related causes. On the contrary, among the 20 pelvic recurrence patients, 25% died due to uncontrolled loco-regional disease, 15% from systemic disease and only 5% from other causes. The cumulative EQD2 dose, toxicity rates and UVA/MVA were not reported.

The final and most recently published retrospective report comes Sunnybrook Health Sciences Centre, Canada, including 39 EC vaginal recurrences, all of whom received SRT from 2014 to 2021, and from the brachytherapy perspective, all patients underwent interstitial BT (ISBT) [21]. This cohort included higher-risk patients (Stage I: 66% and low-grade: 77%) which was reflected on the higher rate of prior adjuvant RT (23%). As a result, more patients received ISBT as the sole treatment (23%) instead of combined EBRT/ISBT (77%), yet in the combined treatment group, all but one were RT-naïve before SRT. This cohort also had a substantial proportion of regional relapses (20%) at the time of vaginal recurrence. The molecular status of the tumours was available (retrospective pathology review not performed) for 10 patients: Five were mismatch repair deficient (MMRd). Five had a p53 mutation (p53abn). One had multiple classifiers (MMRd and p53abn). The median cumulative EQD2 dose of CTV-D90 was 76.8 Gy for the combined treatment and 57.9 Gy for the ISBT group. With a median follow-up of 22 months, at 2 years, actuarial local failure (LF) was 22.6%, while 2-year distant failure was 36.9%, and 2-year OS was 85%. The relatively high LF rate was attributed to the higher-risk patients included in this cohort, though no UVA/MVA was performed. Grade 3 toxicity presenting with rectal bleeding, SBO, soft-tissue necrosis and vaginal stricture was documented in three patients (four events, 10%). No grade 4–5 toxicity was reported even in patients who were retreated with ISBT due to a second recurrence.

Finally, Klopp et al. from the MD Anderson Cancer Centre, Houston, TX, USA, published recently in the *Journal of Clinical Oncology* (JCO) the highly anticipated randomised control trial GOG 0238 focusing on the impact of the addition of concurrent cisplatin to SRT for EC patients with vaginal recurrence [22]. A total of 156 patients were randomised to receive SRT alone (control arm) vs. concurrent cisplatin–SRT (experimental arm). A total of 86% were isolated vaginal recurrences, and 82% were of a low histological grade. With a median follow-up of 62 months, 3-year PFS was 73% for the control arm versus 62% for the experimental arm. The chemoradiation group was associated with higher rates of ≥Gr3 acute toxicity (57% vs. 31%). The investigators concluded that, for vaginal recurrences of EC with a low histological grade, radiotherapy alone remains the best possible treatment option.

**Table 1:** Summary of the literature on EC vaginal recurrence patients who received SRT from 2000 onwards.

Study, Publication Year	Years	Patients (n)	Median Fu (Months)	Pelvic Rec. *	Adj. RT	Stage I	Gr 1–2	Survival Outcomes	Toxicity
Lee 2013 [13]	2003–2011	44	24	14%	30%, EBRT: 16%	73%	73%	2y LC 96%	G3: 3% **
								2y OS 80%	
								2y DFS 72%	
Vargo 2014 [14]	2004–2013	41	18	20%	0%	83%	78%	3y LC 95%	G3: 5%
								3y OS 67%	
								3y DFS 68%	
Chapman 2017 [15]	2000–2010	30	76.4	0%	0%	86%	77%	5y LRFFS: 87%	G5: 1 pt
								5y OS 77%	G3–4: 0
								5y PFS 75%	
Arden 2020 [16]	2004–2018	28	21	18%	0%	93%	82%	2y LC 93%	G3–5: 0
								2y OS 88%	
								2y DFS 80%	
Sapienza 2020 [17]	2009–2018	30	53	7%	3%, VBT: 3%	63%	67%	5y LC 89%	G3: 6%
								5y OS 83%	
								5y DFS 69%	
Lindemann 2021 [18]	2006–2011	139	79	0%	0%	75%	NR ^1^	5y DFS 65%	NR ^1^
								5y OS 68%	
Ørtoft 2024 [20]	2005–2012	141	109	15%	0%	NR ^1^	NR ^1^	NR^1^	NR ^1^
Sherwood 2024 [21]	2014–2021	39	22	20%	23%, EBRT: 13%	66%	77%	2y LF 22.6%	G3: 10%
								2y OS 85%	
								2y DFS 36.9%	
Klopp 2024 [22]	2008–2020	156	62	14%	NR ^1^	NR ^1^	82%	3y PFS 73% ^2^	G3: 31% ^2^
		74 RT							G3: 57% ^3^
		82 CRT							

* Pelvic recurrence (± vaginal) at presentation, ** RT-naïve, and ^1^ NR: not reported. ^2^ RT arm (control) result and ^3^ Cisplatin-RT arm (experimental). LC: local control, LF: local failure, LRFFS: loco-regional failure-free survival, OS: overall survival, DFS: disease-free survival, PFS: progression-free survival, and G: grade.

## 4. Discussion

Modern salvage radiation therapy offers excellent local control rates for this rare group of early-stage, endometrial cancer patients presenting with loco-regional recurrence. In our review, local control ranged from 77.4% to 96%, which is higher compared to historical controls [23,24,25]. Upon conducting the literature review, only one study, by Petignat et al., reported a higher LC rate (100%), but at the cost of significant Gr3–4 toxicity (68%) [26]. Factors associated with local failure were the size of the vaginal recurrence, a high histological grade, non-endometrioid histology and distal vaginal recurrence. Due to the limited number of events, no MVA regarding local control was reported in any of the studies, while UVA was limited; therefore results should be interpreted with caution. For example, distal vaginal recurrences identified in the Harvard study occurred more frequently in high-risk, irradiated patients in the adjuvant setting, leading to lower salvage doses at recurrence due to rectal and urinary toxicity (anatomically closer to distal vagina).

Factors associated with improved DFS are combined EBRT/VBT treatment compared to EBRT or VBT alone and a cumulative EQD2 dose. Combined EBRT/VBT and a cumulative EQD2 dose are both treatment-related factors that can potentially be modified. In Lindemann’s study, based on MVA, combined treatment led to reduced rates of a second relapse and risk of death. Vaginal recurrence patients should have combined treatment when possible, and a cumulative EQD2 dose range from 75 to 85 Gy is recommended in accordance with the Groupe Européen de Curiethérapie–European Society for Radiotherapy and Oncology (GEC-ESTRO) and American Brachytherapy Society (ABS) guidelines [27].

Improved outcomes, especially in local control, are mainly attributed to advances in radiation therapy techniques for both EBRT and brachytherapy. IMRT, compared to 3D-conformal, improves target coverage with adequate doses and spares organs at risk (OARs), therefore decreasing toxicity. This was shown in the post-operative setting by NRG/RTOG 1203 and can be extrapolated at the recurrence setting [28]. IMRT also allows for dose escalation in nodal recurrences. This is common practice in patients with node-positive cervical cancer treated with definitive chemoradiation, as it improves pelvic control [29]. This is particularly important in endometrial cancer, as pelvic control appeared suboptimal in the Ørtoft study, in which a 3D technique was used. On the contrary, in the Vargo study (90% IMRT), nodes were boosted, leading to higher 3-year OS (33%) compared to historical nodal controls, with no Gr3–5 toxicity [30,31]. Brachytherapy has significantly evolved with the use of high-dose-rate (HDR) afterloaders and axial planning (CT and MRI), allowing for target adaptation based on the response to EBRT (image-guided adaptive brachytherapy) and the sparing of OARs. The wider use of interstitial catheters has allowed for extreme dose escalation in complex vaginal targets (asymmetry, size and location far from the vaginal surface) while minimising the dose to OARs owing to brachytherapy’s steep dose gradient. In our review, extremely high rates of interstitial brachytherapy use were reported in three institutions (87%, 90% and 100%, Table 2), giving the opportunity in one of them (Sherwood) to successfully re-irradiate a second recurrence in patients, with no Gr4–5 toxicity. Toxicity reporting is of paramount importance as the majority of patients have a very good prognosis and therefore may experience the potential side effects of salvage radiotherapy. In this review, toxicity was not reported in two studies, including 43% of the total sample. Late genitourinary and gastrointestinal Gr3–5 toxicity rates in the seven studies with toxicity reports were lower compared to historical controls [25,26,32,33].

Cumulative EQD2 dose reporting is another important parameter, reflecting advances in radiation oncology. The cumulative EQD2 dose is the total dose that is intended to be delivered through combined EBRT and VBT, considering the α/β ratios of endometrial cancer (α/β: 10) and OARs (α/β: 3). EQD2 dose reporting of targets and OARs is critical when assessing the quality of radiation treatment planning and delivery as it is predictive of local control and toxicity rates. Data are derived from cervix cancer, where definitive chemoradiation and IGABT are commonly employed in loco-regional disease [34,35,36]. ESGO and ESTRO have set specific targets as quality indicators to standardise, audit and improve the clinical practice of radiation therapy [37]. Cumulative EQD2 D90-CTV (minimum dose delivered to 90% of the clinical target volume) indicates with greater accuracy the dose actually delivered instead of a simple summation of EBRT and VBT, according to the intended doses. In our review, the EQD2 dose was reported in most cases, whereas D90-CTV was reported in four institutions, reflecting the dynamic steps made in endometrial cancer: a higher EQD2 dose was associated with improved LC and DFS, underlining its importance in radiotherapy dose prescription and reporting.

Clinicopathologic factors known to increase the first loco-regional relapse in EC patients, such as advanced age, histological grade, non-endometrioid histology, myometrial invasion >50% and advanced FIGO stage, were also associated with worse survival outcomes following SRT in EC recurrence patients. More specifically, despite the high local control rates demonstrated above, distant relapse is the leading cause of failure, limiting the overall SRT rates. Disease-free survival, distant control, secondary relapse rates and overall survival were all negatively impacted by these factors. In Sapienza’s cohort, one quarter of patients developed distant metastases. In Chapman’s study, there was a statistically significant difference in distant failure-free survival even among stage IA vs. IB patients (100% vs. 50%). In Lindemann’s study, secondary relapse rates increased from 20% in low-risk patients to 69% in high-risk patients. In Vargo’s study the primary mode of failure was distant recurrence (3-year DF rate: 39%), and the anticipated salvage rates for OS were significantly lowered when including patients with higher primary risk stratification than the PORTEC-1 criteria (96% vs. 72%).

Since the anticipated outcomes of SRT seem to be limited by the presence of risk factors, it would be difficult to support an omission of the RT strategy in the adjuvant setting for patients with loco-regional risk factors. Although survival seems unaffected with the omission of RT—as reported in the PORTEC-1 observation cohort and the Nordic studies—it appears that adjuvant vaginal brachytherapy is a safe, efficient and cost-effective treatment choice for most of these patients, without the significant negative impact on the quality of life that comes with EBRT. Adjuvant EBRT should be limited to patients with risk factors for pelvic relapse, such as substantial lymphovascular space invasion (LVSI) or cervical stromal invasion, as pelvic relapses have been shown in our report to carry a worse prognosis compared to isolated vaginal ones [38].

The use of chemotherapy with salvage RT was highly variable in the retrospective cohorts (9–70%) and was not associated with improved outcomes. This was confirmed in the randomised study by NRG, showing that concurrent cisplatin has no place in the context of low-grade isolated vaginal EC recurrence. Its role has yet to be addressed in higher-risk patients (pelvic-nodal recurrence, high grade), along with novel agents such as immunotherapy which has become the new standard of care in the advanced, recurrent and metastatic settings [39,40,41]. Immunoradiotherapy has not been studied in the loco-regional relapse setting, posing new challenges in clinical decision making and underlining the unmet need for more robust data from prospective trials on the safety and efficacy of the combination treatment for this group of patients.

**Figure 2:**
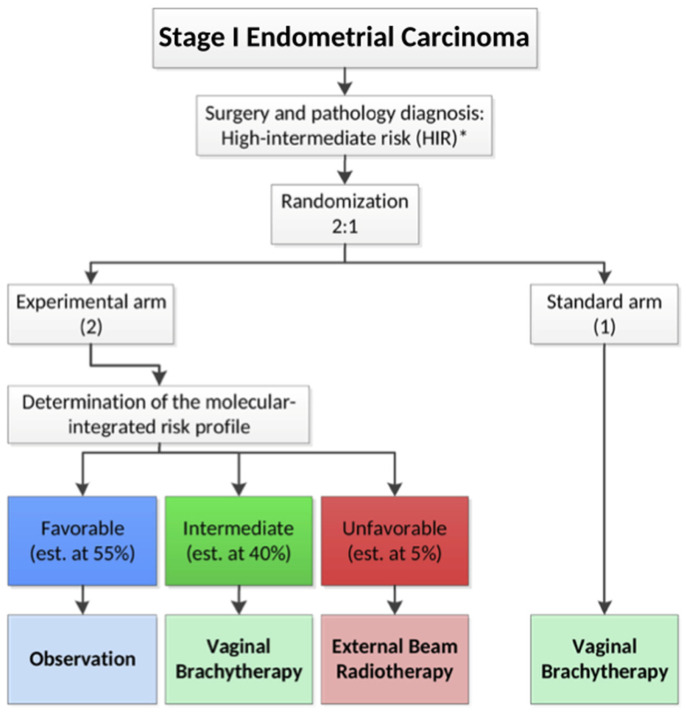
PORTEC-4a study design diagram. * High-Intermediate risk endometrial cancer.

Molecular status [polymerase-ɛ (POLE) mutation, mismatch repair (MMR) status and p53 mutation] has also emerged as an invaluable prognostic and predictive biomarker in endometrial cancer and has been incorporated in the new FIGO 2023 stage and ESGO/ESTRO/ESP risk classification groups. In our review, only 10 patients from one institution had the molecular status reported, making it impossible to associate status with outcomes. Future studies should aim for molecular status to be reported and assessed for potential corelations. The PORTEC-4a trial is a randomised control trial of intermediate–high-risk EC patients, stratifying them in risk groups based on the molecular status and guiding adjuvant treatment accordingly (Figure 2). Mutated POLE and MMR deficiency/wild-type β-catenin are classified as low risk and are allocated to the observation arm. A non-specific molecular profile (NSMP) and MMR-deficient/β-catenin mutation tumours are classified as intermediate risk and are allocated to the VBT arm, whereas mutated p53, substantial LVSI or >10% L1 cell adhesion molecule (L1CAM) expression of the tumours is classified as high risk and is allocated to the EBRT arm [42]. Molecular status should be incorporated in future trials focusing on endometrial cancer loco-regional recurrence patients along with novel treatment agents and local radiotherapy. 

The limitations of our review were mostly related to the retrospective nature of the studies included. This makes it difficult to reach hard conclusions (no MV analysis was performed in most reports due to limited events) or quantify the effects. However, due to the rarity of endometrial cancer recurrence events, it is difficult to overcome this problem, making clinical decision a real challenge in everyday practice. In addition, despite the excellent local control reported in all studies, survival rates were not reported in two studies, making it more difficult to assess the effect of salvage radiotherapy on overall survival. More robust data are necessary to further quantify the effect of SRT on survival. On the other hand, our report focused specifically on early-stage, endometrial cancer recurrence patients receiving modern SRT, as reflected by the recent publication year range (2013–2024), and the results were consistent with improved local control rates and favourable toxicities compared to historical controls.

## 5. Conclusions

Salvage radiation offers excellent local control rates in early-stage, endometrial cancer patients presenting with isolated vaginal recurrence. Survival outcomes post-SRT are limited by the presence of well-known risk factors for the first relapse, with distant control being the primary mode of failure. At present, the decision to omit adjuvant RT should be weighed against the anticipated survival outcomes in case of relapse, with vaginal brachytherapy being the treatment of choice in most cases in the adjuvant setting. Ongoing research incorporating molecular status and focusing on combination treatment with novel agents and salvage RT is of paramount value to address the high distant failure rates and improve survival.

## Figures and Tables

**Table 2:** Summary of salvage RT characteristics.

Study	EQD2 Dose (Gy) D90-CTV (Gy)	IMRT/VMAT	Interstitial IGABT
Lee 2013 [13]	75.5 D90-CTV: 74.8	NR ^1^	80%
Vargo 2014 [14]	76 D90-CTV: 76	90%	27%
Chapman 2017 [15]	68.25 D90-CTV: 70.83	NR ^1^	90%
Arden 2020 [16]	67.6 NR ^1^ as D90-CTV	29%	3%
Sapienza 2020 [17]	EQD2 used, NR ^1^	41%	0%
Lindemann, 2021 [18]	70 NR ^1^ as D90-CTV	NR ^1^	NR ^1^
Ørtoft 2024 [20]	NR ^1^ as EQD2 or D90-CTV	0%	11%
Sherwood 2024 [21]	76.8 ^2^ D90-CTV: 76.8	NR ^1^	100%
Klopp 2024 [22]	Intended dose 75Gy NR ^1^ as D90-CTV	NR ^1^	12%

^1^ Not reported, ^2^ Combined EBRT/BT group, EQD2: equivalent dose in 2 Gy fractions, D90-CTV: minimum dose delivered to 90% of the clinical target volume, IMRT: intensity-modulated radiotherapy, VMAT: volumetric arc therapy, and IGABT: image-guided adaptive brachytherapy.

## Data Availability

No new data were created.
